# Sickle cell disease 115 years later: improving health outcomes through policy, research, and collaboration, to achieve health equity

**DOI:** 10.3389/frhs.2026.1842019

**Published:** 2026-06-03

**Authors:** Emmanuel K. Peprah, Joyce Gyamfi, Nafesa Kanneh, Tania Hameed, Deborah Onakomaiya, Naheed Ahmed, Henny Billett, Andrew Campbell, Amy Cohen, Torian Easterling, Jamillah Hoy-Rosas, José A. Pagán, John Patena, Melinda R. Rushing, Nana Osei-Tutu, Sharee Turpin, Kusum Viswanathan, Angela Odoms-Young, Gbenga Ogedegbe

**Affiliations:** 1 Department of Global and Environmental Health, New York University School of Global Public Health, Implementing Sustainable Evidence-Based Interventions Through Engagement (ISEE) Lab, New York, NY, United States; 2 Institute for Excellence in Health Equity, NYU Grossman School of Medicine, New York, NY, United States; 3 Department of Population Health, NYU Grossman School of Medicine, New York, NY, United States; 4 Montefiore-Einstein Medical Center, New York, NY, United States; 5 Children’s National Hospital, George Washington University School of Medicine and Health Sciences, Washington, DC, United States; 6 The Patient Room, Boston, MA, United States; 7 City College of New York, New York, NY, United States; 8 Ronald McDonald House, New York, NY, United States; 9 Department of Public Health Policy and Management, School of Global Public Health, New York University, New York, NY, United States; 10 NYU Steinhardt, New York, NY, United States; 11 Rutgers, The State University of New Jersey, Edward J Bloustein School of Planning and Public Policy, New Brunswick, NJ, United States; 12 NYC Health+Hospitals/Kings County, New York, NY, United States; 13 University of Rochester, Rochester, NY, United States; 14 One Brooklyn Health, Brooklyn, NY, United States; 15 Division of Nutritional Sciences, Cornell University, Cornell, NY, United States

**Keywords:** health Equity (MeSH), health policies and all other topics, patient centered care, quality of life, sickle cell disease

## Abstract

Sickle cell disease (SCD) has been documented for more than 115 years, yet its scientific and clinical history extends far deeper, beginning with African physicians such as Dr. James Africanus Horton, who described symptoms consistent with SCD decades before its formal recognition in Western medicine. The first modern clinical report, published by Dr. James B. Herrick in 1910, initiated a century of discovery that transformed SCD into the first fully elucidated “molecular disease.” Advances in diagnostics, especially newborn screening, comprehensive care, and treatments, including penicillin, prophylaxis hydroxyurea, stem cell transplant and gene therapies, have transformed hematology and improved survival and quality of life. However, access to these advances remains uneven, reflecting persistent inequities that disproportionately impact SCD communities domestically and globally. Against this backdrop, the SCD 115 Years Later Symposium, held November 12, 2025, explored three interconnected pillars shaping the future of SCD care: research, collaboration, and policy. Four sessions illuminated structural and clinical challenges across the lifespan, emphasizing poor access to care, the need for more holistic care models, the importance of engaging community-based organizations, optimizing and expanding SCD surveillance systems, and stronger policy alignment at state and federal levels. Central themes included unified advocacy, improved care transitions, expansion of multidisciplinary care models, improved access to emerging therapies, and the integration of mental health and psychosocial support into clinical practice. Collectively, the symposium-derived priorities connected scientific progress, policy innovation, and community leadership to improve outcomes for all individuals living with SCD and their families.

## Historical overview of sickle cell disease research over 115 years

The story of sickle cell disease (SCD) spans more than a century of scientific discovery, medical innovation, and human resilience (1, 2). Its earliest roots trace back to Africa, where local physicians and healers observed symptoms consistent with what would later be recognized as SCD. Among the first documented accounts was from Dr. James Africanus Horton, a Sierra Leonean physician and one of the earliest African medical doctors in the British Empire who published, *The Diseases of Tropical Climates and Their Treatment*, in 1872. Horton described a condition resembling sickle cell anemia—decades before the term or its molecular basis was known (3). His pioneering observations, grounded in African clinical realities, represent a critical yet often overlooked foundation in the history of SCD research (4, 5).

Formal recognition of SCD in Western medicine, specifically the United States, occurred nearly four decades later. In 1910, Dr. James B. Herrick, a Chicago-based physician, published the first case report describing “peculiar elongated and sickle-shaped red blood corpuscles” in a patient suffering from severe anemia (6). That patient, Walter Clement Noel, a 20-year-old dental student from Grenada, had presented with recurrent pain crises and respiratory complications—now understood as hallmark manifestations of vaso-occlusive crises (7, 8). Herrick's intern, Dr. Ernest E. Irons, was among the first to observe Noel's distinctive crescent-shaped red blood cells under the microscope (9). This singular observation marked the birth of modern hematology's understanding of SCD and catalyzed over a century of global research. Despite chronic illness, Noel completed his studies and returned to Grenada to practice dentistry, exemplifying remarkable perseverance, though he ultimately succumbed to complications of the disease at age 32 (7). Noel's legacy endures as both the first documented case in the medical literature and as a symbol of the countless patients whose experiences have driven scientific discovery and compassionate care in SCD.

## Scientific milestones and therapeutic evolution of SCD research

Over the past century, SCD research has advanced through a series of landmark discoveries. In 1933, scientists in Memphis conducted a study of over 2,500 African Americans, distinguishing between sickle cell trait and SCD, clarifying its genetic inheritance (10). The identification of sickle hemoglobin as the first “molecular disease” by Linus Pauling and colleagues in 1949 revealed that an abnormal hemoglobin structure underlies its pathology, ushering in a molecular era for SCD research (11). Subsequent discoveries illuminated the broader biological context: in 1954, the protective effect of sickle cell trait against malaria was established, explaining the prevalence of the gene in malaria-endemic regions (12). A diagnostic blood test developed in 1955 enabled reliable identification of abnormal hemoglobin, and by 1957, the molecular defect—a single amino acid substitution—was pinpointed, making SCD the first genetic disorder with a fully elucidated molecular basis (2, 13).

The 1960s and 1970s marked both scientific and policy breakthroughs. x-ray crystallography revealed the three-dimensional structure of hemoglobin in 1960, earning Max Perutz the Nobel Prize in 1967 (14, 15). In 1972, the National Sickle Cell Anemia Control Act, championed by Dr. Roland Scott, established screening, counseling, education, and research programs across the United States (16). New York State led the way in implementing mandatory newborn screening in 1975 (17), laying the foundation for early detection and longitudinal care. Therapeutic innovations soon followed: in 1986, an National Heart, Lung, and Blood Institute (NHLBI)-funded study demonstrated that prophylactic penicillin reduced life-threatening *Streptococcus pneumoniae* infections by 84% in children, transforming pediatric care (18). Hydroxyurea, validated in the 1995–1998 Multicenter Study and the 2011 BABY HUG trial, became the first Food and Drug Administration (FDA)-approved pharmacologic therapy to reduce pain crises and hospitalizations (19, 20). L-glutamine, was subsequently approved in 2017 to reduce acute complications of sickle cell disease (21). Most recently, December 2023 saw the FDA approval of two gene therapies—Casgevy (CRISPR/Cas9-based) and Lyfgenia (viral vector-mediated)—ushering in an era of potentially curative, one-time treatments (22, 23). Despite these advances, challenges remain in ensuring broad access to these innovations, particularly among historically marginalized communities disproportionately affected by SCD (24–26). Persistent implementation gaps, resource constraints, provider shortages, and structural inequities hinder the widespread uptake of evidence-based interventions across the lifespan.

Recognizing these challenges, we—along with many others in the field—have called for intentional and sustained multi-sector collaboration to accelerate implementation and strengthen comprehensive SCD care (27–31). The SCD 115 Years Later Symposium emerged directly from these calls to action. It was motivated by the recognition that scientific breakthroughs alone are insufficient without coordinated efforts among clinicians, researchers, policymakers, patients, caregivers, and community advocates. The Symposium represents a collective commitment to aligning voices, sharing evidence, and advancing solutions that bridge scientific discovery with real-world impact.

## Why advancing SCD health outcomes is essential: research, collaboration, and policy

The SCD 115 Years Later Symposium, hosted on November 12, 2025 on the New York University Medical Campus, emphasized three interconnected themes—research, collaboration, and policy—highlighting both achievements and persistent gaps in health equity (32). The themes underscored the historical trajectory from the discovery of sickle hemoglobin to modern gene therapies, revealing the long delay between identification and therapeutic innovation. While hydroxyurea and gene-based therapies represent major breakthroughs, disparities in research funding, clinical trial participation, and access continue to limit benefits for SCD communities domestically and globally. Collaboration is a critical driver of progress. From early population studies in the 1930s to contemporary global implementation efforts (33), meaningful advances in SCD care and support have relied on collaboration and partnerships among patients, families/caregivers, clinicians, and researchers. Policy also remains a central pillar in translating research into equitable care. Legislative frameworks, such as the National Sickle Cell Anemia Control Act of 1972, have enabled nationwide screening, education, and research initiatives (13)..

The symposium opened with remarks from Dr. Emmanuel Peprah, who underscored that SCD is both a local and global health condition affecting millions, yet remains particularly urgent to address in New York State, where approximately 10% of all individuals living with SCD in the United States reside—most concentrated within New York City. Dr. Gbenga Ogedegbe followed the opening remarks with a deeply moving personal story that illustrated two critical themes: first, that deaths from SCD represent not abstract statistics but profound losses that reverberate across families and communities; and second, that research funding for SCD remains disproportionately low compared with other conditions, such as cystic fibrosis, despite comparable disease burden. Dr. Michael Merson followed with reflections from his clinical experiences treating patients with SCD in both New York City and Baltimore, emphasizing the interwoven clinical and structural challenges that shape patient outcomes. The opening segment also featured a powerful and poignant video from *The Pitt*, an American television series on the HBO network, depicting a woman during a severe SCD pain crisis, which highlighted the profound human toll of the disease beyond clinical descriptions. Together, these opening perspectives framed the Symposium's call for greater visibility, advocacy, and investment in SCD research and care.

## Approach to synthesis of SCD symposium comments

To ensure a rigorous, transparent, and reproducible process for translating insights from the SCD: 115 Years Later Symposium into a scholarly manuscript, we employed a structured, multi-step qualitative synthesis approach. *1. Selection of Panelists and Participants*. Panelists and participants were purposively selected to ensure diverse and balanced representation across key stakeholder groups, including clinicians, researchers, community advocates, policymakers, and individuals with lived experience of SCD. Each panel intentionally incorporated individuals with lived experience to center patient and community perspectives. Selection criteria prioritized subject-matter expertise, demonstrated leadership in SCD-related work, and the ability to contribute meaningfully to the symposium's core thematic areas (e.g., research, collaboration, and policy). *2. Invitation-Based Structure of the Symposium*. The symposium utilized a curated, invitation-based format to facilitate high-level, focused discussions among experts and stakeholders actively engaged in SCD research, clinical care, and advocacy. This approach fostered in-depth dialogue, encouraged candid exchange of ideas, and ensured alignment with the symposium's objectives. *3. Data Capture and Documentation*. All symposium sessions were systematically documented using detailed note-taking by designated rapporteurs, supplemented by notes from session moderators. The symposium was intentionally not audio- or video-recorded to promote open dialogue—particularly for individuals with lived experience of SCD—thereby reducing concerns related to stigma and enabling participants to share perspectives more freely. Following the symposium, the lead author collected notes from all rapporteurs and moderators and conducted an initial synthesis of the discussions. These summaries were subsequently shared with session moderators to verify accuracy and ensure alignment with the intended themes of each session. *4. Selection of Illustrative Quotes.* Representative quotes were purposefully selected to illustrate key themes and preserve the authenticity of participant perspectives. Quotes were chosen based on their clarity, relevance, and ability to reflect broader thematic patterns, rather than highlighting isolated or anecdotal viewpoints. Minor edits were made where necessary to improve readability while maintaining original meaning. 5*. Panelist Review of Summary Outputs.* To enhance validity and ensure accurate representation, synthesized themes and summary outputs were shared with all panelists and moderators for review and feedback (member-checking). This process allowed participants to confirm the interpretation of their contributions, provide clarification, and strengthen the credibility of the final synthesis.

## Symposium key themes

The symposium's four sessions (Table 1) collectively demonstrated how research, collaboration, and policy converge to advance SCD care 115 years after its initial scientific description by Herrick (6). Each session contributed a unique lens—equity, policy, clinical practice, and partnership—highlighting the multidimensional strategies required to transform outcomes for individuals and families affected by SCD.

**Table 1:** Participant composition and areas of expertise represented across the four panels of the 2025 sickle cell disease (SCD) symposium.

Sessions I- IV: Moderators & Panelists		
Session I		
Moderator	Panelist	Expertise
Naheed Ahmed, PhD, MPH, MA. Assistant Professor, NYU Grossman School of Medicine	DaMia Harris-Madden, Ed.D., M.B.A, M.S. H.R.M., Commissioner, NYS Office of Children and Family Services	New York State Commissioner for Children and Family Services, providing statewide leadership on policies that affect child and family health, well-being, and equity.
	Lewis Marshall, MD. Chief Medical Officer (CMO), NYC Health + Hospitals/Lincoln	
		Emergency Medicine Physician with deep expertise in complex health systems, offering frontline insight into acute care delivery, patient flow, and structural barriers affecting individuals with chronic conditions.
	Kusum Viswanathan, MD, FAAP, CMO, One Brooklyn Health (OBH), Brookdale, Director, Comprehensive Pediatric Sickle Cell Program-OBH, Professor of Clinical Pediatrics SUNY- Downstate Health Sciences University	
		Pediatric Hematologist with more than 40 years of clinical experience in sickle cell disease, recognized for lifelong commitment to caring for children and adolescents with SCD across New York State.
	Nana Osei-Tutu, MSc, SCD Patient and Advocate Infection Preventionist NYC Health + Hospitals/Kings County	
		SCD Patient Advocate with lived experience, bringing critical perspectives on patient-centered care, stigma, and the real-world challenges faced by individuals and families navigating SCD.
Session II		
Moderator	Panelist	Expertise
José A. Pagán, PhD. Professor and Chair, NYU School of Global Public Health	Kenneth Rivlin, MD, PhD. Vice Chair, Dept. of Pediatrics, NYC Health + Hospitals/Jacobi	SCD Clinician with more than 30 years of experience providing comprehensive care to individuals living with SCD across New York State, with deep expertise in both acute and longitudinal management.
	Ravi Singh Vice President, Market Access, Advocacy & Policy, Genetix Biotherapeutics	
		Industry Leader in Emerging Genetic and Curative Therapies, offering specialized knowledge in the development, evaluation, and implementation of next-generation treatments for SCD.
	Melinda Rushing, PhD, LMSW Assistant Professor, Rutgers University	
		SCD Researcher Advancing Health System Innovation, focused on designing and testing tools, decision-support strategies, and implementation approaches to improve outcomes and reduce disparities within healthcare systems.
	Amy Cohen, MPH SCD Patient and Advocate	
		SCD Patient Advocate with lived experience, bringing essential insights into patient needs, real-world care challenges, and the importance of centering the patient voice in policy and practice.
Session III		
Moderator	Panelist	Expertise
Torian Easterling, MD. Senior Fellow for the Health and Opportunity Leadership Institute (HOLI) at CCNY’s Colin Powell School for Civic and Global Leadership and Director for Young Doctors Project-NY	Henny H. Billett, MD. Director, Hemophilia Treatment Center, Montefiore/Albert Einstein College	SCD Clinician with more than 30 years of experience, providing comprehensive, lifespan-spanning care for individuals living with SCD across New York State, and serving as a leading voice in clinical excellence and care delivery improvement.
	Toni Eyssallenne, MD, PhD. Deputy Chief Medical Officer, NYC Department of Health and Mental Hygiene	
	Sharee Turpin SC Patient Navigator, University of Rochester Medical Center (URMC) Golisano Children Hospital	
		Senior SCD Physician with over three decades of practice, recognized for advancing evidence-based management, mentoring the next generation of SCD providers, and shaping clinical standards within the New York City healthcare system.
		SCD Patient Advocate with lived experience, offering essential insights into patient needs, healthcare navigation challenges, and the importance of integrating the patient voice into clinical, research, and policy decision-making.
Session IV		
Moderator	Panelist	Expertise
Angela Odoms-Young PhD. Associate Professor, Cornell University	Jamillah Hoy-Rosas, MPH, RD. Head of Population Health, Ronald McDonald House–NYC	Expert in health equity and community linkage, specializing in connecting underserved populations to essential healthcare services, strengthening care coordination, and addressing structural barriers that impede access.
	Andrew D. Campbell, MD. Director, Comprehensive Sickle Cell Center & Associate Professor, Children’s National Hospital & George Washington University	
		SCD clinician with more than 30 years of experience in New York State, recognized for delivering comprehensive care across the lifespan and advancing clinical best practices for individuals living with SCD.
	Maia Z. Laing, Chief Policy Officer, Sick Cells	
	Ifeyinwa (Ify) Osunkwo, MD, MPH, Chief Patient Officer for Rare Disease, Novo Nordisk, Professor of Medicine & Pediatrics, Maya Angelou Research Center for Healthy Communities (affiliate)	
		Legislative and policy specialist, SCD patient advocate, and individual with lived experience, bringing a dual perspective that informs equitable policy development, patient-centered reforms, and community-engaged advocacy.
		Senior SCD physician with over three decades of clinical practice in the United States, contributing national expertise in disease management, system-wide quality improvement, and the implementation of evidence-based approaches to SCD care.

**Session I**, moderated by Naheed Ahmed, PhD, MPH, MA centered on *Health Equity in Sickle Cell Disease*, illuminating the persistent disparities that shape clinical outcomes, access to care, and patients' lived experiences. Panelists (see Table 1) emphasized that advancing equity requires coordinated, multisector collaboration among clinicians, community-based organizations (CBOs), policymakers, and patient advocates to dismantle longstanding systemic barriers. Key priorities included strengthening patient and provider education on SCD's clinical course, variability, and treatment options, and improving provider training to reduce stigmatizing and mislabeling of patients as drug-seeking. The session also highlighted the fragile transition from pediatric SCD care to adult SCD care, calling for models of care (34–36) that ensure continuity of services as adolescents enter early adulthood (37–41). Mental health (42, 43) and psychosocial support emerged as essential to holistic care, prompting calls to integrate social workers, community health workers, and peer navigators to link patients with community-based social needs support related to mental wellbeing, housing, food, and education. These roles were viewed as critical bridges between clinical care and community-based resources, particularly for patients navigating recurrent hospitalizations and complex social determinants of health.

This theme was powerfully illustrated by the following reflection from a panelist:

“As part of my assignment at Kings, I work in the outpatient hemodialysis unit. For our renal failure patients, they are constantly battling with the symptoms and treatment side effects of their condition. As a necessary part of their care, social workers play an important role, particularly when it comes to psychosocial support. I recall having my first crisis at the age of 17, and I remember how scared I was at the time because it was my first ever hospitalization. My parents were also very worried, and I think at the time, it would have been great for not only me, but my parents, to have similar access to a social worker. There needs to be a greater push for more holistic care for sickle cell patients.”

This quote highlights key gaps in SCD care by contrasting it with renal care, where psychosocial support is routinely integrated, underscoring its inconsistent availability for SCD patients. The panelist's personal experience illustrates the emotional vulnerability surrounding early hospitalizations, particularly during adolescence, and the missed opportunity for early psychosocial intervention to support both patients and families. Collectively, the reflection reinforces the need to institutionalize patient and family-centered psychosocial services as a core component of comprehensive and equitable SCD care.

The panelist identified Policymakers as critical actors in expanding funding for SCD clinical trials as well as protecting and strengthening federal and state policies that promote overall well-being and quality of life for patients with SCD. Programs such as Supplemental Nutrition Assistance Program (SNAP) (44–47), the Affordable Care Act (ACA) (48–50), Medicaid/Medicare (51, 52), were identified as critical safety nets that directly influence patients' access to care, treatment adherence, and overall quality of life. In addition, speakers underscored the importance of sustained investment in CBOs, which often serve as trusted sources of education, advocacy, and psychosocial support for individuals and families affected by SCD (53–55). Complementing these policy priorities was a call for more comprehensive and inclusive data systems that integrate clinical outcomes with patient-reported experiences to inform equitable health system transformation. Collectively, these perspectives anchored Session I in an equity-driven framework that emphasized structural change alongside clinical innovation.

**Session II**, led by José A. Pagán, PhD, focused on Policy Solutions and underscored the essential role of legislation and regulatory frameworks in shaping SCD outcomes. Speakers reviewed current initiatives at both the state and national levels. In New York State, panelists (see Table 1) identified opportunities to better align laws with the needs of health systems, communities, and SCD patients. A central theme was the importance of enhancing communication and coordination across the SCD community, insurers, healthcare systems, community-based organizations, and policymakers to ensure a unified approach to treatment access and resource allocation. The session also emphasized strengthening care transitions during adolescence and young adulthood, recommending the expanded use of health homes (56, 57), case management (58, 59), and community health workers (60–62) to support navigation and adherence. Participants highlighted the need for clear dissemination of emerging therapies so that patients, families, and payers are prepared as treatment options evolve. At the state-level, participants pointed out the value of coordinated policy efforts—including policy mandates such as stigma-reduction training in emergency departments—to enhance patient experience and health outcomes. Through this lens, Session II established policy alignment as a mechanism for enabling systemic change.

**Session III,** moderated by Torian Easterling, MD, focused on *Enhancing Care Delivery Across the Lifespan* and underscored the need for coordinated, evidence-based strategies that support individuals with SCD from childhood through older adulthood. Discussion emphasized the importance of ensuring seamless continuity of care across pediatric, adult, and geriatric services, recognizing that the needs of aging patients with SCD are evolving and often underserved. Participants (see Table 1) highlighted the need to improve early detection through enhanced provider and family training, as well as delivering tailored education for clinicians, parents, and caregivers at moments that align with clinical workflows. The session also addressed caregiver burnout (63–65) and called for targeted support systems to alleviate emotional and logistical burdens. Recommendations included streamlining hospital workflows to better integrate SCD care, improving testing turnaround time, and ensuring timely communication of results and diagnoses. Strengthening relationship-building between institutions, providers, patients, and families was identified as central to improving trust and care coordination. Multidisciplinary teams were encouraged to conduct bi-weekly case reviews to reinforce accountability and best practices. Additional priorities included expanding reproductive care education for patients with SCD, enhancing pain management within emergency departments, and leveraging New York City Department of Health initiatives—such as public health detailing—to engage providers and bolster system-wide collaboration. Session III ultimately advanced a vision of lifelong, integrated, whole person care as essential for transforming how health systems advance care for people living with SCD.

**Session IV**, moderated by Angela Odoms-Young, PhD, focused on *Linking Policy to Practice Through Collaborations and Partnerships* and demonstrated how multisector engagement can advance policy and generate sustainable, patient-centered solutions. Panelists (see Table 1) emphasized that the impact of policy depends on equitable and effective implementation at the local, community, and systems levels. They also noted that strong policies can sustain investment in CBOs that provide essential services and advocacy, improve SCD care by expanding coverage, and support innovative care and payment models (66), such as the Centers for Medicare & Medicaid Services (CMS) New York Health Equity Reform (NYHER) 1115 Waiver Demonstration Amendment, which directs funding to social care networks to address health-related social needs. Similarly, discussions also illuminated how shifts in Medicaid eligibility, SNAP benefits, and other policy changes (e.g., ACA) directly affect patient and family stability. Developing a unified advocacy agenda emerged as essential for mobilizing communities and ensuring consistent, coherent messaging across sectors. The session also highlighted the need to ensure equitable access to comprehensive, culturally responsive care that addresses both clinical and social needs, supported by an expanded SCD workforce (30, 67)—including community health workers, peer navigators, hematologists, and general practitioners. Finally, speakers stressed the importance of centering lived experience in decision-making and elevating the voices of SCD warriors in shaping policy and practice. Session IV thus articulated a partnership-based model for translating policy into meaningful, sustainable practice.

## Aligning priorities and policy to improve SCD care

Across the four symposium sessions, several overarching themes emerged that collectively underscored the need for unified action to advance SCD care, research, and policy (see Table 2). A central theme was the importance of speaking with one voice. Panelists emphasized that, similar to the HIV/AIDS community, a unified advocacy agenda has the potential to accelerate progress in securing national and state-level legislation that meaningfully improves outcomes for SCD patients. The HIV/AIDS movement was cited as a model for how coordinated messaging, shared priorities, and sustained community engagement helped drive historic policy and funding advances (68, 69). Closely linked to this theme was the need for strategic priority setting. Participants highlighted that aligning clear, consensus-driven priorities would strengthen the SCD community's collective voice and enhance its ability to influence policy. These priorities must reflect both national and state-level needs, ensuring that legislative and regulatory actions address structural gaps across the continuum of SCD care.

**Table 2:** Synthesis of symposium recommendations generated by panelists and participants at the 2025 sickle cell disease (SCD) symposium, highlighting strategic priorities across research, policy, and care delivery.

Table 2: SCD 115 Years Later Next Steps
Create a shared advocacy plan so communities, providers, patients, and organizations can speak with a stronger, united voice to influence policies and funding. Join existing coalitions or working groups focused on SCD policy and align messaging and priorities. Increase long-term funding for comprehensive sickle cell programs to enable them to provide the much-needed social work services within the program office. Identify and apply for federal, state, and private grants that support care coordination and social work services. Continue to support community-based organizations (CBOs) and community health workers who serve and support people with SCD. Partner with local CBOs to co-host education sessions, support groups, or screening events. Shift research focuses from quantitative measures (demographic data, ED utilization, etc.) to qualitative measures (experiences with stigma, knowledge of the condition, etc.), for a more mixed-method approach to understanding the condition. Collaborate with CBOs to co-design surveys and interview guides that reflect lived experience. Build fair, community-driven data systems that ensure information about SCD reflects real experiences and is used to improve services and accountability. Establish community advisory boards to guide what data are collected and how results are shared. Strengthen partnerships across health care and social service systems so families receive support not only for medical and mental health needs but also for challenges related to food, housing, transportation, and insurance. Formalize referral pathways with social service agencies, food banks, housing organizations, and transportation programs. Make sure people living with SCD are at the center of decision-making by creating paid leadership and advisory roles that value their knowledge and experience. Create compensated patient and caregiver advisory councils for clinics, health systems, and research projects. Advocate for strengthening comprehensive care models that integrate services provided by various specialists (i.e., hematology, psychology, cardiology, nephrology, etc.) ensuring patients receive coordinated, holistic care rather than fragmented treatment across multiple providers, and strive to reduce implicit bias in pain management. Provide regular training for clinicians on SCD guidelines, implicit bias, and evidence-based pain management.

At the national level, panelists discussed two major pieces of federal legislation reintroduced in 2024 aimed at strengthening the SCD care infrastructure. The Sickle Cell Disease Treatment Centers Act (S. 5226/H.R. 9872) proposes establishing a national network of comprehensive SCD treatment centers using a hub-and-spoke model, with central expert centers providing oversight, technical assistance, and support to smaller community-based sites (70). Complementing this effort, the Sickle Cell Disease Comprehensive Care Act (S. 996/S. 5097) focuses on creating Medicaid “health homes” to deliver coordinated, multidisciplinary outpatient care for SCD, particularly those covered by Medicaid. Both bills seek to improve access, care coordination, and quality of life SCD patients; however, despite their reintroduction in 2024, neither has yet passed the U.S. Congress. Currently, Sickle Cell Disease Comprehensive Care Act (S.721) was re-introduced in the U.S. Senate by Senator Cory Booker at the 119th Congress (2025-2026) (71). This legislation can begin to address the disparities encountered by the SCD community by funding coordinated care teams, improving support for care transitions (especially from pediatric to adult care), expanding access to evidence-based therapies, and strengthening data systems that track outcomes and gaps in care—changes that are particularly critical for adolescents and young adults with SCD (67).

In addition to federal legislation, panelists highlighted the importance of national level policy initiatives such as the CMS Cell and Gene Therapy (CGT) Access Model, which holds significant implications for the SCD community. As of 2024, 33 states, along with the District of Columbia and Puerto Rico, are participating in this national demonstration project aimed at expanding access to high-cost genetic therapies, including recently FDA approved treatments for SCD (38). The model employs outcomes-based agreements, linking reimbursement to measurable improvements in patient health outcomes and thereby encouraging both affordability and accountability in the delivery of gene-based treatments (72). By reducing financial barriers and incentivizing effective care, the CGT Access Model represents a critical step toward more equitable access to transformative therapies (73–75).

## Limitations

This output of the symposium should be interpreted in light of several limitations. First, the findings are derived from a single symposium, which, while comprehensive, does not constitute a systematic or exhaustive review of all available evidence or perspectives in SCD. Second, because panel participation was invitation-based, this may have limited the inclusion of certain viewpoints, particularly from underrepresented regions or populations. Third, the symposium discussions were largely centered on New York State, which has a distinct healthcare infrastructure and policy environment. Although many themes are broadly relevant, the generalizability of specific recommendations to other U.S. regions or global settings may be limited. Finally, no formal consensus methodology (e.g., Delphi process) was used nor were patient-level outcome data presented. As such several policy and programmatic issues discussed were time-sensitive and may evolve, particularly in areas such as healthcare policy and emerging therapies. Despite these limitations, this work provides a timely synthesis of multidisciplinary perspectives to inform future SCD research, policy, and implementation efforts.

## Conclusion

The SCD 115 Years Later Symposium reaffirmed that, despite extraordinary scientific progress and recent breakthroughs in disease-modifying and gene-based therapies, SCD patients continue to face profound inequities in access, quality of care, and health outcomes. The discussions highlighted that scientific innovation alone is insufficient without coordinated policy action, targeted and sustainable funding, and meaningful patient and community engagement. Speakers, moderators, and panelists emphasized the critical importance of unified advocacy—mirroring successful models from the HIV/AIDS movement—to elevate national attention, expand research investment, and drive system-level reforms. Moreover, the SCD Symposium underscored the need to strengthen care delivery across the lifespan, particularly during critical transition periods, while expanding multidisciplinary models that integrate clinical, behavioral, and social care. Personal testimonies reminded participants that SCD is not merely a public health challenge but a deeply human one, marked by stigma, loss, resilience, and the urgent need for patient-centered solutions. Collectively, the symposium called for an aligned, equity-driven agenda that bridges research, policy, and community leadership to ensure that scientific advancements translate into sustained meaningful, measurable improvements in the lives of all individuals and families affected by SCD.

## Data Availability

The raw data supporting the conclusions of this article will be made available by the authors, without undue reservation.
